# Impairment of viral replication capacity by *nef* alleles from HIV elite controllers

**DOI:** 10.1186/1742-4690-8-S2-P53

**Published:** 2011-10-03

**Authors:** Philip Mwimanzi, Tristan Markle, Hirohito Otsuka, Yoko Ogata, Michiyo Tokunaga, Toshiyuki Miura, Eric Martin, Florencia Pereyra, Bruce Walker, Zabrina Brumme, Mark Brockman, Takamasa Ueno

**Affiliations:** 1 Center for AIDS Research, Kumamoto University, Kumamoto, Japan; 2 Simon Fraser University, Burnaby, BC, Canada; 3 University of Tokyo, Tokyo, Japan; 4 Ragon Institute of Massachusetts General Hospital, Boston, MA, USA

## Background

Elite controllers (EC) are HIV-1 infected persons who can spontaneously control viremia to extremely low levels. Because it is known that *nef is* required for HIV-1 replication and pathogenesis *in vivo*, we wish to clarify how HIV-1 *nef* plays a role in acquiring EC phenotype.

## Materials and methods

Viral RNAs were extracted from plasma samples of 45 ECs and 48 chronic progressors (CP) and used for amplification of *nef* by RT-PCR. The amplified products were cloned into a plasmid and subsequently sequenced. A representative *nef* clone was selected for each patient and cloned into an NL43 proviral plasmid backbone. Infectious recombinant viruses were prepared by transfecting 293T cells with these resultant proviral clones and tested for their viral replication capacity in PBMCs obtained from multiple healthy donors. Viral replication was monitored by measuring the concentration of p24 Gag in the culture supernatant by p24 ELISA every 3 days. The same virus stocks were further tested for their infectivity using TZM-bl cells.

## Results

All *nef* alleles isolated from EC and CP had intact ORFs. Phylogenetic analysis revealed no specific lineage or clustering, suggesting the absence of common genetic defects in EC-derived *nef* alleles. All viruses expressing patient-derived *nef* showed a wide range of viral replication in PBMC, confirming the importance of *nef* alleles in the enhancement of viral replication capacity. The viruses harboring EC-derived *nef* replicated and peaked on Day 12 after infection or even later; whereas the viruses harboring CP-derived *nef* peaked on Day 9. Also, the peak level of p24 values was significantly lower in the EC-*nef* viruses than in the *CP-nef* viruses. Because Nef activity is highly dependent on PBMC donors, we tested 4 different donors' PBMC and compared the initial burst of their viral replication at Day 9. In all donors'PBMC, viruses carrying EC-*nef* displayed lower p24 values than those carrying *CP-nef*, suggesting that the viral replication capacity was much impaired by *nef* alleles in EC. Corroboratively, in viral infectivity assay in TZM-bl cells, the EC-*nef* viruses showed significantly lower infectivity than the CP-*nef* viruses.

## Conclusion

Taken together, our data indicate that *nef* alleles present in circulating plasma viruses in EC are much impaired in boosting viral replication, suggesting that such phenotypic alterations in Nef function play a role, at least partly, in the sustained maintenance of low viral load *in vivo.*

